# Orchid Phylotranscriptomics: The Prospects of Repurposing Multi-Tissue Transcriptomes for Phylogenetic Analysis and Beyond

**DOI:** 10.3389/fpls.2022.910362

**Published:** 2022-05-27

**Authors:** Darren C. J. Wong, Rod Peakall

**Affiliations:** Ecology and Evolution, Research School of Biology, The Australian National University, Canberra, ACT, Australia

**Keywords:** phylogeny, orchids, transcriptome, next-generation sequencing–NGS, plastome, target sequence capture sequencing, phylogenomics, phylotranscriptomics

## Abstract

The Orchidaceae is rivaled only by the Asteraceae as the largest plant family, with the estimated number of species exceeding 25,000 and encompassing more than 700 genera. To gain insights into the mechanisms driving species diversity across both global and local scales, well-supported phylogenies targeting different taxonomic groups and/or geographical regions will be crucial. High-throughput sequencing technologies have revolutionized the field of molecular phylogenetics by simplifying the process of obtaining genome-scale sequence data. Consequently, there has been an explosive growth of such data in public repositories. Here we took advantage of this unprecedented access to transcriptome data from predominantly non-phylogenetic studies to assess if it can be repurposed to gain rapid and accurate phylogenetic insights across the orchids. Exhaustive searches revealed transcriptomic data for more than 100 orchid species spanning 5 subfamilies, 13 tribes, 21 subtribes, and 50 genera that were amendable for exploratory phylotranscriptomic analysis. Next, we performed re-assembly of the transcriptomes before strategic selection of the final samples based on a gene completeness evaluation. Drawing on these data, we report phylogenetic analyses at both deep and shallow evolutionary scales *via* maximum likelihood and shortcut coalescent species tree methods. In this perspective, we discuss some key outcomes of this study and conclude by highlighting other complementary, albeit rarely explored, insights beyond phylogenetic analysis that repurposed multi-tissue transcriptome can offer.

## Introduction

The Orchidaceae is rivaled only by the Asteraceae as the largest plant family, with an estimate of some 25,000 species (∼ 8% of plants) shared across at least 700 genera (Chase et al., 2015). Orchids can be found in a wide range of habitats across tropical, subtropical, and temperate regions (Swarts and Dixon, 2009), and hold great interest from an ecological and evolutionary standpoint. They are particularly well known for their diverse morphological adaptations such as epiphytism, with at least 18,000 species adopting this lifestyle (Gravendeel et al., 2004), and their dependence on mycorrhizal fungi interactions for germination (Phillips et al., 2020). Additionally, many orchids employ highly specialized or unusual pollination strategies (Jersáková et al., 2006; Schiestl and Schlüter, 2009; Bohman et al., 2016; Wong et al., 2017b; Trunschke et al., 2021). These characteristics make orchids ideal targets for exploring diverse ecological and evolutionary questions (Peakall, 2007).

### Plastome and Mitochondrial Genome Phylogenomics in Orchids

To gain insights into the mechanisms driving the species diversity of the Orchidaceae across both global and local scales, investigations targeting different taxonomic and geographic scales (e.g., across specific tribe to genus) and a well-supported phylogeny are crucial. Rapid advancements in high-throughput sequencing and bioinformatics methods have enabled the use of genome (nuclear, plastid, and mitochondrial) and transcriptome sequences (including coding and non-coding regions) amendable for phylogenetic inference (McKain et al., 2018). Currently, the newfound ease of sequencing whole plastomes makes cpDNA the most widely used target for both small- and broad-scale orchid phylogenetic studies in terms of size (i.e., from tens to hundreds of species) and taxonomic breadth (i.e., within a genus or across five subfamilies) (Barrett et al., 2014; Givnish et al., 2015; Li Y. X. et al., 2019; Kim et al., 2020; Liu et al., 2020; Smidt et al., 2020; Serna-Sánchez et al., 2021). Notably, the first broad-scale plastome phylogeny for the Orchidaceae was based on maximum-likelihood (ML) analysis of 75 cpDNA coding-genes across 39 genera representing all five subfamilies and 16 (of 17) tribes (Givnish et al., 2015). Many previously underrepresented genera are now included in a cpDNA study of 78 genes spanning 264 species, 117 genera, 18 tribes, and 28 subtribes (Serna-Sánchez et al., 2021).

### Phylotranscriptomics in Orchids

Notwithstanding the extraordinary insight from plastome studies gained in the last decade, phylogenetic studies leveraging transcriptome datasets to identify putative single-copy genes (or orthogroups) are now emerging as a new tool to aid plant molecular systematics. Compared to the linked genes in cpDNA which collectively represent just one evolutionary history, nuclear genes offer several advantages including their biparental inheritance, higher substitution rates, and access to thousands of unlinked genes each with the potential to represent independent evolutionary histories, among others (Zhang et al., 2012; Smith, 2015).

To date, several broad-scale transcriptome-based phylogenetic analyses of the Orchidaceae have been performed, but with limited taxon sampling compared to the available plastome-based phylogenies (Deng et al., 2015; Zhang et al., 2017; Guo et al., 2018; Unruh et al., 2018; Piñeiro Fernández et al., 2019; Wong et al., 2019). Nonetheless, one study incorporated the transcriptomes of 13 orchid species representing five subfamilies and 10 phylogenetic informative non-orchid outgroups to build a high-confidence phylogenetic tree of the Orchidaceae from >700 single-copy orthogroups using both concatenation and coalescence-based summary methods (Unruh et al., 2018). This analysis firmly placed the genus *Cypripedium* sister to the rest of the slipper orchid genera (e.g., *Phragmipedium*, *Mexipedium*, *Paphiopedilum*, *Selenipedium*), a previously uncertain relationship. The effectiveness of using transcriptomes to resolve relationships at shallower scales have also been demonstrated. For example, previously uncertain phylogenetic relationships among closely related orchid species (e.g., 8 *Cypripedium*, 4 *Ophrys*, and 5 *Gymnadenia* spp.) have also been clarified using hundreds of putative single-copy genes (Guo et al., 2018; Piñeiro Fernández et al., 2019).

### Target Sequence Capture Phylogenomics in Orchids

Putative single-copy genes are already being used to design target enrichment probe sets capable of spanning broad evolutionary scales. Two universal probe sets that provide orchid coverage are: Angiosperms353 (Johnson et al., 2019) and Orchidaceae963 (Eserman et al., 2021). For example, a recent phylogenomic analysis of 75 species representing 69 genera, 16 tribes, and 24 subtribes used the Angiosperms353 universal probe set to target 294 low-copy nuclear genes (Pérez-Escobar et al., 2021). This study revealed that higher-level phylogenetic relationships were robust and largely congruent with earlier plastome- and mitochondrial genome-based phylogenies (Li Y. X. et al., 2019; Serna-Sánchez et al., 2021). However, several instances of strongly supported discordances in both shallow and deep time were also revealed. The adaptability of universal probe sets, such as Angiosperms353, for resolving within-genus relationships of some *Epidendrum* (Granados Mendoza et al., 2020) and *Lepanthes* (Bogarín et al., 2018) orchids, has also been demonstrated. Nonetheless, the use of more targeted probe sets may be necessary to provide greater phylogenetic resolution at the subtribe to subspecific levels, as exemplified by a study of 30 genera spanning the tribe Diurideae and 24 closely related *Caladenia* species (Peakall et al., 2021).

### Opportunities for Repurposing Transcriptomes for Phylogenetic Analysis in Orchids

In this perspective, we demonstrate that rapid and accurate phylogenetic insights across the Orchidaceae can be gained from repurposed multi-tissue transcriptomes that were sequenced primarily for non-phylogenetic studies. We conclude by discussing some feasible avenues of research beyond phylogenetic analysis that repurposed multi-tissue transcriptome can offer. In 2021, we conducted an exhaustive search for orchid transcriptome datasets in public repositories and the literature. Our search revealed transciptomes were available for more than 100 orchid species spanning 5 subfamilies, 13 tribes, and 20 subtribes, and drawn from over 50 published studies (Supplementary Table 1).

To use this large volume of data effectively, it was necessary to first re-assemble the transcriptomes from the publically accessible raw data. In total, the transcriptomes of 133 target orchid species were reassembled from a total of ∼ 8.5 billion high-quality reads using *Trinity* (Haas et al., 2013). Next, the outcomes of a gene completeness evaluation using BUSCOs (Waterhouse et al., 2018) was used to gauge assembly quality and to cull samples with poor scores (i.e., < 60%). Finally, generic level representation was restricted to a maximum of two (when available) species per genus (Supplementary Figure 1 and Text). Thus, our final high-quality orchid-wide phylotranscriptome data set contained 69 orchids (48 genera) spanning 5 subfamilies, 13 tribes, and 21 subtribes. Four non-orchid Asparagales species (i.e., *Molineria capitulata*, *Hypoxis hemerocallidea*, *Lanaria larata*, *Borya sphaerocephala*) were included as outgroups. Single-copy (strict and relaxed) phylogenetic-informed orthogroups were then identified using OrthoFinder (Emms and Kelly, 2019) and concatenated alignments of protein sequences were evaluated by maximum-likelihood and shortcut-coalescent phylogenetic analysis using IQTREE (Minh et al., 2020) and ASTRAL (Zhang et al., 2018), respectively (see Supplementary Text and Methods for additional details).

### Taxonomic Representation Across the Phylotranscriptome Data Set

**FIGURE 1:**
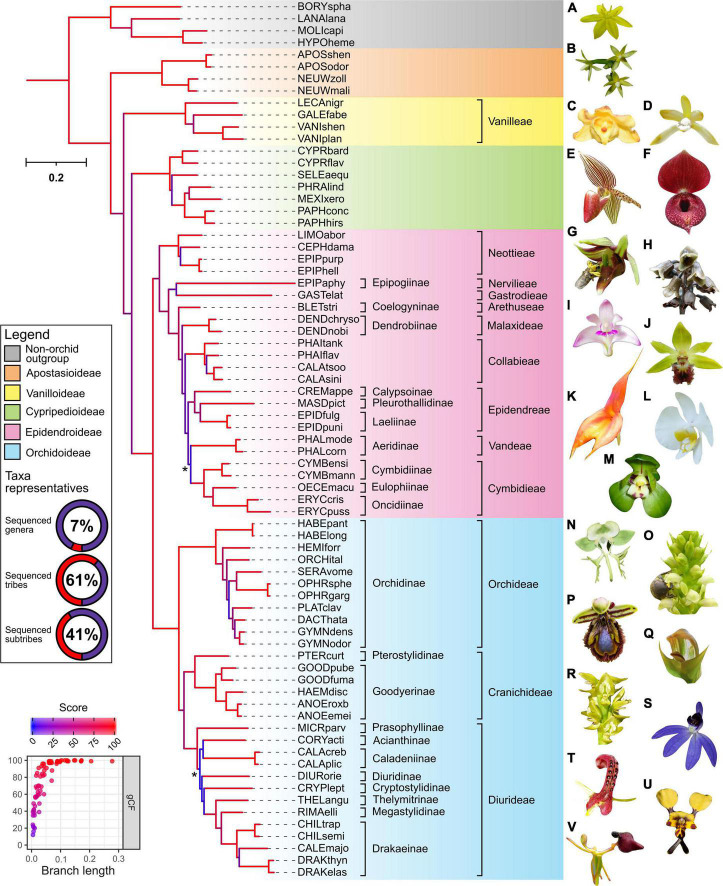
Outcomes of phylogenetic analysis across the Orchidaceae from repurposed multi-tissue transcriptomes. The maximum-likelihood IQ-TREE phylogeny of 69 orchids (spanning 5 subfamilies, 13 tribes, 21 subtribes, and 48 genera) and four non-orchid species as outgroup was based on partition analysis of 633 amino-acid alignments with a total of 317,221 (139,913 parsimony-informative) sites (see Supplementary Methods for details). The abbreviated species name is shown (see Supplementary Table 1 for details). Background color indicates subfamily grouping. The donut chart depict the taxonomic representativeness of included orchid genera, subtribe, and tribe (see relevant placeholder in the phylogeny for details). Asterisk indicate ultrafast bootstrap (UFboot) or SH-aLRT branch support <95 and <80%, respectively. Branch length indicates the total number of substitutions per site and branch colors correspond to gene concordance factors (gCF)–the proportion of gene trees that decisively support the given bifurcation. Inset (bottom left) shows a strong positive relationship of gCF scores with branch length. See Supplementary Figure 2 for additional details on the corresponding shortcut coalescent ASTRAL phylogeny. Representative species of the outgroup **(A)** *Hypoxis hirsuta* and subfamilies across the Orchidaceae are indicated **(B)** *Apostasia nipponica*–Apostasioideae; **(C)** *Galeola lindleyana* and **(D)** *Lecanorchis suginoana*–Vanilloideae; **(E)** *Paphiopedilum rothschildianum* and **(F)** *Cypripedium lichiangense*–Cypripedioideae; **(G)** *Epipactis veratrifolia*, **(H)** *Gastrodia fontinalis*, **(I)** *Dendrobium kingianum*, **(J)** *Calanthe tricarinata*, **(K)** *Masdevallia veitchiana*, **(L)** *Phalaenopsis amabilis*, and **(M)** *Cymbidium goeringii*–Epidendroideae; **(N)** *Habenaria limprichtii*, **(O)** *Satyrium microrrhynchum*, **(P)** *Ophrys speculum*, **(Q)** *Pterostylis curta*, **(R)** *Prasophyllum elatum*, **(S)** *Cyanicula caerulea*, **(T)** *Cryptostylis leptochila*, **(U)** *Diuris pardina*, and **(V)** *Drakaea glyptodon*–Orchidoideae. All images have been reproduced with permission from the respective copyright holders. Please refer to the section “Acknowledgments” for image credits.

Despite originating from public databases which constrains the choice of samples available, the final phylotranscriptome data set of 69 species achieved wide taxonomic coverage across the Orchidaceae and outgroups (Figure 1). This included all known genera of the Apostasioideae and Cypripedioideae subfamilies, while only the tribe Pogonieae lacked representation within the Vanilloideae. Tribes spanning both the lower and higher Epidendroideae were also well covered, with a total of 16 genera, 9 tribes, and 10 subtribes represented. Finally, despite a trend of the Orchidoideae being previously under-represented, this gap has been filled in this study. In particular, genome-scale phylogenetic studies (based on whole plastome, transcriptome, or mitochondria genome) have had poor representation of the diverse Australasian tribe the Diurideae (Givnish et al., 2015; Li Y. X. et al., 2019; Kim et al., 2020; Smidt et al., 2020). Here our phylogenetic analysis included 18 Diurideae species from 10 genera and 8 subtribes. Consequently, this phylotranscriptome dataset provides one of the most comprehensive genera and subtribe representations for the Orchidoideae in phylogenomic studies to date, with a total number of genera, tribes, and subtribes of 22, 3, and 11, respectively (Figure 1).

### Deep and Shallow Phylotranscriptome Relationships Are Robust and Consistent With Other Phylogenomics Datasets

The outcomes of our phylotranscriptomic analysis (Figure 1) revealed robust and largely consistent findings with other phylogenomics datasets (Givnish et al., 2015; Kim et al., 2020; Liu et al., 2020; Eserman et al., 2021; Pérez-Escobar et al., 2021; Serna-Sánchez et al., 2021). As expected, the backbone phylogeny of the *Orchidaceae* revealed five distinct clades: Apostasioideae (4 species) was placed sister to the other four subfamilies, followed by Vanilloideae (4 species), and by Cypripedioideae (7 species) which is placed sister to Orchidoideae (30 species) and Epidendroideae (24 species). Within the Vanilloideae subfamily, *Lecanorchis* was placed sister to a clade formed by *Galeola* and *Vanilla*. Within the Cypripedioideae, *Cypripedium* is basal, followed by *Selenipedium* successively sister to a clade formed by *Phragmipedium*, *Mexipedium*, and *Paphiopedilum* species.

Within the Epidendroideae, for almost all branches leading to the relevant tribes except *Vandeae*, *Epidendreae*, and *Cymbidieae*, the topology was also consistent with other orchid-wide species trees inferred from genome-scale datasets. For example, clear separation between lower (i.e., tribes Neottieae, Gastrodieae, Nervilieae) and higher (i.e., in the order of tribes Arethuseae, Malaxideae, Collabieae, Epidendreae, Vandeae, and Cymbidieae) Epidendroid species were evident. The only case of poor branch support, low gCF and sCF (i.e., proportion of decisive gene trees and alignment sites supporting a given branch supporting a given branch, respectively), and local PP scores pertains to the placement of the tribe Vandeae (i.e., *Phalaenopsis* spp.) in the ML and ASTRAL species tree (Supplementary Figure 2 and Text for further information). This point of uncertainty is consistent with findings in other recent broad-scale nuclear orchid phylogenomic studies (Eserman et al., 2021; Pérez-Escobar et al., 2021). This now well characterized disparity is postulated to reflect widespread incomplete lineage sorting of nuclear genes during the rapid radiations at the early stages of the evolution of the Vandeae, Epidendreae, and Cymbideae tribes (Eserman et al., 2021).

Within the Orchidoideae, the tribe Orchideae (11 species) is placed sister to a clade formed by Diurideae (13 species) and Cranichideae (6 species) with robust support. Subtribe relationships within Cranichideae were resolved with the subtribe Goodyerinae forming a well-supported clade, sister to *Pterostylis* (subtribe Pterostylidinae). Relatively short branch lengths and low gCF/sCF scores along the backbone and some terminal taxa of the tribes Diurideae and Orchideae were also observed. Within Orchideae, the genus *Habenaria* was the outermost group followed by the genus *Hemipilia* and *Orchis*. Next, the genus *Serapias* forms a clade with *Ophrys* and is placed sister to a clade containing *Platanthera*, *Dactylorhiza*, and *Gymnadenia* species.

Concerning the tribe Diurideae, most subtribe and genus level relationships were congruent with those obtained from a recent phylogenomic study of this tribe using a multi-tiered sequence capture strategy (Peakall et al., 2021). Like the former study, poor support and very low gene concordance factors were observed on the very short but deep branch that separates the sole outermost genus, *Microtis* (Prasophyllinae) from the rest of the tribe. As in the Epidendroideae, such uncertainly likely reflects the incomplete lineage sorting during the rapid radiations underpinning the formation of the major subtribes (Peakall et al., 2021).

To ascertain if robust phylogenetic relationships can be attained at shallower evolutionary depths (i.e., within specific subfamily and genus) from repurposed transcriptomes, samples corresponding to the Cypripedioideae subfamily (19 species) and the genus *Phalaenopsis* (11 species) were targeted. Almost all nodes showed robust support and the topologies closely mirror those inferred using a few chloroplast and low-copy nuclear genes but with broader taxon sampling (Tsai et al., 2010; Guo et al., 2012). As already indicated by other phylotranscriptomic studies (Guo et al., 2018; Unruh et al., 2018; Piñeiro Fernández et al., 2019), our analyses re-confirm the potential for the phylogenetic analysis of transcriptomes to resolve relationships at relatively shallow scales.

### The Prospects of Using Transcriptomic Datasets for Phylogenetic Inference and Beyond

While our study has convincingly demonstrated the potential of phylotranscriptomic studies across the Orchidaceae, there are still several major challenges posed across multiple levels. For example, despite our recent exhaustive search of orchid transcriptomes in public repositories, comprehensive coverage spanning: i. many genera, tribes, and subtribes (Li Y. X. et al., 2019; Kim et al., 2020; Serna-Sánchez et al., 2021) or ii. expanded coverage within a particular genus (Guo et al., 2021) is lacking when compared to those from recent plastome phylogenies. Thus, given the size of the Orchidaceae, we recommend targeted sequencing to help fill obvious gaps not yet available in the public datasets. Nonetheless, some phylotranscriptome studies demonstrate that past transcriptomic datasets can be a key resource for increasing taxonomic breadth, which when lacking is known to affect overall phylogenetic accuracy (Zwickl and Hillis, 2002). One example is the phylotranscriptome of Asterids where approximately 40% of the 365 species represented were largely repurposed (Zhang et al., 2020).

In the same way that transcriptomes can be utilized for phylogenetic studies, assembled whole genome sequences may be repurposed for combined phylogenomic/transcriptomic studies of the Orchidaceae. For example, high-depth whole-genome sequencing of 689 vascular plant species from a botanical garden (Liu et al., 2019) contained at least 10 orchid species spanning the genera *Dendrobium*, *Renanthera*, *Vanda*, *Tropidia*, and *Hylophila*, among others, may be used to complement the phylotranscriptomic analysis presented here.

In the Orchidaceae, mycoheterotrophy has evolved sporadically with estimates of some 200 almost fully mycoheterotrophic species, spanning 43 genera (Merckx, 2013). Partial mycoheterotrophy is also likely to be much more widespread across the orchids, than currently documented (Gebauer et al., 2016). Fully mycoheterotrophic orchids often exhibit severely reduced plastid genome, thus, they are often devoid of adequate plastid gene sequences for phylogenetic analysis (Feng et al., 2016). Partially mycoheterotrophic orchid species may also exhibit varying degrees of plastome degradation. In such cases, nuclear DNA sequences may provide the only reliable data for robust phylogenetic analysis (Cameron, 2004; Górniak et al., 2010; Freudenstein and Chase, 2015). Our findings from transcriptomic data show that the placement of *Gastrodia elata* is consistent with earlier orchid-wide phylotranscriptome studies (Deng et al., 2015) while the relationship of *Epipogium aphyllum* (tribe Nervilieae) being sister to *G. elata* (tribe Gastrodieae) appears to be a new finding from this present study. Interestingly, the exceptionally long branch lengths in the clades formed by the mycoheterotrophic *G. elata* and *E. aphyllum* also points to accelerated substitution rates of nuclear genes at a genome-wide scale, a general feature of many parasitic plants (Bromham et al., 2013).

The difficulty in scaling up standard phylogenetic procedures to hundreds, if not thousands of species, will likely persist in future orchid phylotranscriptome studies. Therefore, we take the opportunity to highlight one promising approach using homologous *k*-mer (substring of sequence) blocks that bypass several arduous steps including annotation, ortholog detection, and alignment (Sanderson et al., 2017). Interestingly, Sanderson et al. (2017) recovered trees using homologous *k*-mer blocks and published ones were nearly identical in all seven genome-scale datasets tested containing tens to hundreds of species highlighting its robustness and scalability. Preliminary evaluation of this approach on target sequence capture data encompassing hundreds of loci and samples–67 samples spanning the tribe Diurideae and 72 samples involving 24 closely related *Caladenia* species–revealed its potential to recover species trees matching conventional workflows but also better group morphologically similar members of species complexes (Peakall et al., 2021).

Past and emerging transcriptome datasets can also be mined for the Angiosperms353 (Johnson et al., 2019) and Orchidaceae963 single-copy genes (Eserman et al., 2021) for downstream phylotranscriptomic analysis. This approach bypasses the need for the arduous ortholog inference that often scales poorly with large datasets spanning hundreds of species. However, it remains to be seen if these predefined single-copy genes will be effective in resolving shallow and/or complex evolutionary relationships (e.g., in recent and/or rapid radiating lineages). For the latter purpose, the use of thousands of single-copy genes unique to the study system enabled by targeted studies may be more effective as exemplified in this present study (Supplementary Figures 3, 4) and several others (Guo et al., 2018; Wu et al., 2018; Piñeiro Fernández et al., 2019; Züst et al., 2020; Peakall et al., 2021).

### Benefits of Phylotranscriptomics for Gene Duplication and Selection

The prospects of using transcriptomic datasets to gain insights into whole-genome (WGD) or lineage-specific gene duplication events (Zhang et al., 2017; Unruh et al., 2018) and genome-wide detection of positively selected genes of specific evolutionary branches (Balao et al., 2017; Guo et al., 2018; Wong et al., 2019) are also promising avenues of research. For example, two studies have identified a single WGD event shared by all (extant) orchids using transcriptomes from a largely independent list of orchid and non-orchid species (Zhang et al., 2017; Unruh et al., 2018). Lineage-specific gene duplication events leading to the diversification of Cypripedioideae have also been inferred and mapped onto the phylogeny (Unruh et al., 2018).

Signatures of genes under positive selection that may have contributed to species diversification or adaptive evolution in specific evolutionary branches of orchids have also been revealed. For example, elevated evolutionary rates at several candidate volatile biosynthesis-related genes (e.g., fatty acid metabolism) have been independently observed in the unrelated genera of *Chiloglottis* and *Cypripedium* (Guo et al., 2018; Wong et al., 2019). In *Chiloglottis*, the genes encoding the β-ketoacyl-ACP synthase homologs implicated in the biosynthesis of the 2,5-dialkyl cyclohexane-1,3-dione volatiles (chiloglottones) that attract the specific male wasp pollinators (Peakall et al., 2010), show strong signatures of selection (Wong et al., 2019). In two *Dactylorhiza* orchids, positively selected genes were notably enriched with biotic defense-related pathways related to physical and chemical adaptations (Balao et al., 2017).

## Conclusion

In this study, we showcase for the first time that a large set of publically available transcriptomes that were sequenced primarily for non-phylogenetic studies can be effectively repurposed to gain phylogenetic insights across broad evolutionary scales of the Orchidaceae. This strategy greatly increased the genus, tribe and subtribe representation of both small (e.g., Cypripedioideae) and larger subfamilies (e.g., Orchidoideae and Epidendroideae) and provided access to thousands of informative sequences (e.g., putative single-copy genes or orthogroups) amendable for phylogenetic inference. We predict that phylotranscriptomic studies will provide an additional platform for future molecular systematic studies and other investigations into the mechanisms driving the extraordinary species diversity of the Orchidaceae.

## Data Availability Statement

The datasets presented in this study can be found in online repositories *via* NCBI Sequence Read Archive (SRA) accession from respective publications listed in Supplementary Table 1. When records of the original publication(s) are not available, the designated Bioproject accession (PRJNA#) is shown.

## Author Contributions

DW designed the study and analyzed the data. RP and DW secured funding. DW wrote the manuscript with assistance from RP. Both authors have read and approved the manuscript.

## Conflict of Interest

The authors declare that the research was conducted in the absence of any commercial or financial relationships that could be construed as a potential conflict of interest.

## Publisher’s Note

All claims expressed in this article are solely those of the authors and do not necessarily represent those of their affiliated organizations, or those of the publisher, the editors and the reviewers. Any product that may be evaluated in this article, or claim that may be made by its manufacturer, is not guaranteed or endorsed by the publisher.
