# Supplementary material for: Orchid Phylotranscriptomics: The Prospects of Repurposing Multi-Tissue Transcriptomes for Phylogenetic Analysis and Beyond
Source: Front Plant Sci. 2022 May 27;13:910362. doi: 10.3389/fpls.2022.910362 (PMC9196242; doi:10.3389/fpls.2022.910362)
Supplement: Supplementary file 2 [file Data_Sheet_1.pdf]

SUPPLEMENTARY TEXT

Orchid phylotranscriptomics: The prospects of repurposing multi-tissue transcriptomes for phylogenetic analysis and beyond

Darren C.J. Wong[1*] and Rod Peakall[1]

[1]Ecology and Evolution, Research School of Biology, The Australian National University, Canberra ACT 2600, Australia

*** Correspondence:**

Darren CJ Wong

darren.wong@anu.edu.au; wongdcj@gmail.com

+61 2 6125 9892

Compilation of publicly available orchid transcriptomes

Exhaustive searches for orchid transcriptome datasets in public repositories and literature revealed more than 100 species spanning 5 subfamilies, 13 tribes, and 21 subtribes drawn from more than 50 published studies. Several more transcriptomes were unpublished but are publicly-available (**Table 1**). Briefly, the majority of studies often contained just a few (1 – 2) representative species from a specific subfamily, tribe, or subtribe. Nonetheless, 7 studies (6 published, 1 unpublished) contributed a total of 66 species spanning 5 subfamilies, 8 tribes, and 14 subtribes. Finer-scale efforts targeting multiple species from a specific tribe, subtribe, or genus were also relevant (e.g. 18 species from the tribe Diurideae of the Orchidoideae subfamily). A summary of species used in this study according to their known subfamily, tribe, and subtribe designations is listed in **Table 1**.

As assembled transcriptomes for the majority of target species were not readily available and multiple sequencing strategies and tissue types were used, we streamlined the assembly process by selecting sequencing libraries from either leaf- and/or floral-related tissues (when available) for transcriptome re-assembly. As such, the two most predominant tissues used for the assemblies were the leaves and flowers (buds inclusive), each with > 60 spp. represented. Nonetheless, tissues such as stems (5 spp.), whole plant (4 spp.), seeds (3 spp.), mixed tissues (3 spp.), and roots (8 spp.) were also included when the latter three tissue types were not available (**Table 1**). By doing so, we avoided the absolute requirement of using all tissue datasets for a given target species for transcriptome assembly when available (e.g. roots). Thus, significant time-savings in the pre-processing and assembly stage were gained. One recent study showed that despite the risk of missing some plant genes because not all genes are expressed in all plant tissue transcriptomes, phylotranscriptomic trees were fairly robust to the types of tissues used and the strict requirement for a common set of tissue types across all species was not necessary (Cheon et al., 2020). For example, phylotranscriptome trees of 15 vascular plants constructed from one of three available tissues at random from each species (i.e. leaf, root, or stem) were largely consistent with the phylogenomic dataset.

Repurposing multi-tissue transcriptomes for phylogenetic analysis of the Orchidaceae

Transcriptomes of 133 target orchid species were assembled from a total of ~ 8.5 billion high-quality (filtered PE and merged PE reads/super-reads) reads using a single assembler (i.e. *Trinity*). This was the preferred choice as a meta-analysis of several transcriptome assemblers highlighted *Trinity* as one of the top performers for producing consistently good assemblies across datasets from different species and tissue types, among other criteria (Hölzer and Marz, 2019). The preliminary (full) assemblies contained between ~ 30,000 – 500,000 (97,795 median) transcripts, had N50 scores between 0.4 – 3.0kb (1.6kb median), and an average length between 0.4 – 1.5kb (0.9kb median), among others (**Supplementary Figure 1, Supplementary Table 1**). The final set of assemblies were obtained after filtering spurious (e.g. misassembled, fragmented) and redundant transcripts and selection of accurate coding gene sets (see **Supplementary Methods** below). In the final assemblies, transcripts were significantly reduced up to 10-fold (4-fold median reduction) while the distribution of N50 score and average length across each assembly slightly increase compared to the full assemblies.

To further assess the completeness of expected gene content in the assemblies, the orthology status of predicted genes was determined using BUSCO sequence profiles within the embryophyta lineage database (**Supplementary Figure 1**). BUSCO assessments indicated that most final assemblies were generally of high quality, with a median of 92.5%

completeness, despite the subset of tissue(s) used for the assembly. However, there were still a few cases (e.g *Dactylorhiza fuchsii*, *Dactylorhiza incarnata*, and *Oncidium sphacelatum*) with poor completeness coverage (7 – 11%) within the pool of 133 target orchids. After excluding assemblies that failed our criteria, we obtained very robust assembly metrics such as a median N50 and BUSCO gene completeness scores of 1.6kb and 90%, respectively across the 133 species under consideration (**Supplementary Table 1**). Comparable assembly and BUSCO quality metrics between previously assembled orchid transcriptomes (Chao et al., 2017; Piñeiro Fernández et al., 2019) and this study were also observed despite using a smaller subset of available tissues and a different assembly pipeline. Thus, our simplified methodological approach (i.e. selective use of tissues for assembly and a good all-rounded transcriptome assembler) did not compromise the resulting transcriptome quality metrics.

Once robust assemblies are in hand, further downstream success depends on accurate single-copy ortholog identification (Cheon et al., 2020). In plants, numerous phylotranscriptomics studies have successfully resolved deep relationships using putative single-copy orthologs at the family (e.g. Brassicaceae in Huang et al., 2016; Rosaceae in Xiang et al., 2017), order (Zhang et al., 2020), and kingdom level (Wickett et al., 2014; Leebens-Mack et al., 2019). Likewise, most phylotranscriptome studies in orchids to date have employ this approach for understanding relationships at the subfamily level (Deng et al., 2015; Zhang et al., 2017; Unruh et al., 2018; Wong et al., 2019). Of the 133 orchid species, we first prioritised those that have adequate gene completeness (i.e. > 60%) and at most two species with the best BUSCO completeness scores of each genus. To that end, 69 orchids (48 genera) spanning 5 subfamilies, 13 tribes, and 21 subtribes, and 4 non-orchid species as outgroups were selected (**Figure 1, Supplementary Figure 2**).

Maximum-likelihood species tree inference based on amino acid sequences was first attempted using phylogenetic-informed orthogroups identified using *OrthoFinder* (**Figure 1**). This option is renowned for its accurate single-copy ortholog identification and scalability with large genome-scale datasets across hundreds of taxa (Emms and Kelly, 2015, 2019). *OrthoFinder* only identified 78 strictly single-copy orthogroups in the prioritised orchid-wide dataset. While transcriptomes provide a wealth of sequences, not all genes encoded by the genome are necessarily expressed in a given tissue type (i.e. flower vs leaf vs root). This is further compounded by the complexities of plant transcriptomes which are shaped by large-scale gene duplication and loss events, and also the combined effect of increasing species count and divergence times between them (Lee et al., 2013; Emms and Kelly, 2018, 2019; Qiao et al., 2019). These factors are often additive and negatively impact the discovery of phylogenetic-informative markers amendable for species tree inference across wide taxonomic scales, thus, making the identification single-copy orthogroups particularly challenging for plants (Emms and Kelly, 2018, 2019; Cheon et al., 2020). The prioritised orchid-wide dataset illustrates this case where strict single-copy orthogroups were rare (< 100) relative to the tens of thousands of orthogroups identified.

The selection of orthogroups that are single-copy for a proportion of species offers one solution to this problem. The approach offers improved accuracy to concatenated multiple sequence alignments and ASTRAL species tree inference across a wide range of datasets when an adequate number (i.e. > 100) of single-copy orthogroups is lacking (Emms and Kelly, 2018, 2019). Indeed, an additional 271, 555, and 824 relaxed single-copy orthogroups were identified with at most one (r1), two (r2), or three (r3) species having gene duplicates within each selected orthogroup, respectively. Therefore, the corresponding ML species trees

were based on partition analysis of 347 (r1), 633 (r2), and 902 (r3) amino-acid alignments (gapped) from these orthogroups with a total of 177,395 (78,870 parsimony-informative), 317,221 (139,913 parsimony-informative), and 455,502 (202,970 parsimony-informative) sites, respectively. In addition, the partitions ranged between 200 – 2,300 sites (median of 490) and contain between 65 – 72 (median of 71) species represented (See Supplementary Data).

The ML IQTREE phylogeny based on amino acid sequences of the prioritised orchid-wide dataset have identical tree topology across the three levels of the accepted threshold for species having duplicates across orthogroups (i.e. r1 – r3). Lowering this threshold (i.e. r2 vs r1) improved overall branch support slightly but only to a certain point (i.e. branch support was almost identical for r2 and r3 trees). As such, the ML species tree was constructed using 88 strictly and 570 relaxed single-copy orthogroups (r2 total) and discussed here on in. Virtually all but three nodes showed reliable branch support (i.e. SH-aLRT ≥ 80%, UFboot ≥ 95%). However, it is noteworthy that concatenation analyses with phylogenomic datasets often return fully resolved trees (i.e. all branches have 100% support) despite having potentially high levels of underlying gene tree discordance (Kumar et al., 2012). Therefore, we also assessed the underlying disagreement among loci and sites at each node using concordance factors (Minh et al., 2020a). Across the phylogeny, the gene concordance factor (gCF) and site concordance factor (sCF) values were significantly correlated ($R = 0.867$), with an average gCF and sCF of 74 and 63, respectively. The observed distribution of concordance factors (both gCF and sCF) are also highly comparable with other phylogenomic datasets (Minh et al., 2020a).

Inconsistent placement of the tribe Vandeae across various phylogenomics datasets

As expected, the backbone phylogeny of the *Orchidaceae* revealed five distinct clades: Apostasioideae (4 species) was placed sister to the other four subfamilies, followed by Vanilloideae (4 species), and by Cypripedioideae (7 species) which is placed sister to Orchidoideae (30 species) and Epidendroideae (23 species). Relationships between genera within the subfamilies Apostasioideae, Vanilloideae, and Cypripedioideae all received SH-aLRT and UFboot support of 100%. Similarly, the shortcut-coalescent ASTRAL tree constructed using ML gene trees (with very low UFBoot support branches contracted, i.e. < 33%) has a nearly identical topology with the ML IQTREE phylogeny (**Figure 1, Supplementary Figure 2**). Additionally, most nodes showed reliable branch support (localPP of 1) except for a few having very short branch lengths (in coalescent units) and low localPP scores (i.e. < 0.8). These associations somewhat mirror the branch support and length metrics for many relationships observed in the ML tree. The only case of poor branch support, low gCF, sCF, and localPP scores pertains to the placement of the tribe Vandeae (i.e. *Phalaenopsis* spp.) in the ML and ASTRAL species tree.

Interestingly, our results reflect the well known uncertainty of the placement of the Vandeae which varies with the different types of data (e.g. plastid, mitochondrial, and single-copy nuclear genes), selection of genes (e.g. Angiosperms353 and Orchidaceae963), and tree inference approaches used (e.g. ML, ASTRAL, and BI) across several genome-scale phylogenies. For example, most ML and ASTRAL plastome phylogenies indicate the placement of Epidendreae sister to Cymbidieae–Vandeae clade (Kim et al., 2020; Pérez-Escobar et al., 2021; Serna-Sánchez et al., 2021). An alternate topology having Vandeae sister to Cymbidieae–Epidendreae clade is nonetheless indicated with BI tree constructed using the plastome sequences of an entirely distinct set of species indicated Vandeae sister to Cymbidieae–Epidendreae clade (Li et al., 2019) and Orchidaceae963 ASTRAL trees

(Eserman et al., 2021). Similarly, conflicting results supporting the alternate topologies also arise with the choice of genes used – e.g. 252 (Eserman et al., 2021) vs 294 (Pérez-Escobar et al., 2021) genes of the same Angiosperms353 probeset. Here, we also see the same topological conflict with our choice of single-copy genes and phylogenetic inference methods.

Phylogenetic analysis at shallower evolutionary scales: thousands of single-copy orthogroups clarify highly diverse and phylogenetically shallow relationships of the Orchidaceae

Motivated by the promise of resolving deep phylogenetic relationships, we wanted to determine whether robust phylogenetic relationships can also be attained at shallower evolutionary depths (e.g., within specific subfamily, tribe, subtribe, and/or genus) from repurposed transcriptomes. To that end, we focussed on the small Cypripedioideae subfamily (19 species) and one genus with at least 10 representative species, e.g. *Phalaenopsis* (11 species). Unlike the prioritized orchid-wide phylogeny, the difficulty in finding strictly single-copy orthogroups in such small datasets quickly diminishes. For example, the Cypripedioideae-targeted phylogeny (19 species and *Vanilla shenzhenica* as outgroup) is composed of 1,905 strictly single-copy orthogroup alignments and 985,695 total sites (155,901 parsimony-informative sites). Similarly, the *Phalaenopsis*-targeted phylogeny (11 *Phalaenopsis* species and *Erycina pusilla* as outgroup) is composed of 4,453 strictly single-copy orthogroups alignments encompassing 2,748,285 total sites (113,564 total and parsimony informative sites). Across both inferred trees virtually all nodes had 100% SH-aLRT and UFBoot support (**Supplementary Figure 3 and 4**).

Within the the Cypripedioideae phylogeny, the among genus phylogenetic relationships were identical to those observed with the prioritised orchid-wide phylogeny, albeit low gene tree concordance (ca. 39%) supporting the placement of the genus *Selenipedium aequinoctiale* were still observed. Within *Cypripedium*, the phylotranscriptome tree indicated *C. acaule* (section Acaulia) as the outermost group. This is followed by a clade formed by *C. singchii* (section Subtropica) and *C. formosanum* (section Flabellinervia), then by *C. flavum* (section Obtusipetala), then by a clade formed by *C. bardolphianum* and *C. micranthum* (section Sinopedilum), and finally a clade containing four species of section Trigonopedia (i.e. *C. lentiginosum*, *C. margaritaceum*, *C. fargesii*, and *C. sichuanense*). The percentage of gene trees supporting the latter two sections were high (96 and 76%, respectively) but not others (e.g. section Acaulia, Subtropica, Flabellinervia, and Obtusipetala). Within *Paphiopedilum*, *P. malipoense* (subgenus *Parvisepalum*) was basal, followed by *P. concolor* (subgenus *Brachypetalum*), and by a clade (subgenus *Paphiopedilum*) containing *P. callosum*, *P. spicerianum*, *P. henryanum*, and *P. hirsutissimum*. Between- and within-genus phylogenetic relationships are largely consistent with the molecular phylogenetic analysis of this subfamily using a few chloroplast and low-copy nuclear genes (Guo et al., 2012) and those reported from their original phylotranscriptomic studies (Guo et al., 2018; Unruh et al., 2018).

The inferred phylogenetic relationships within the genus *Phalaenopsis* closely mirror those obtained from earlier studies using a few plastid and nuclear loci but with much broader sampling (Tsai et al., 2010). The phylotranscriptome tree indicated two distinct clade containing those belonging to subgenus *Phalaenopsis* (5 species) and subgenus *Polychilos* (6 species) following the classification of a wide extent of *Phalaenopsis* species (Tsai et al., 2010). The proportion of gene trees supporting the bifurcation of subgenus *Phalaenopsis* and *Polychilos* were also reasonably high (64 and 69%). Within the subgenus *Phalaenopsis*, *P. celebensis* was sister to *P. equestris* (section Stauroglottis), followed by a clade (section

Phalaenopsis) formed by *P. schilleriana*, *P. aphrodite*, and *P. amabilis*. Within the subgenus *Polychilos*, *P. cornu-cervi* (section Polychilos) was sister to a clade formed by *P. fasciata*, *P. luddemanniana*, *P. bellina*, *P. javanica*, and *P. modesta* (section Amboinenses).

SUPPLEMENTARY METHODS

De novo transcriptome assembly

Publicly available high-throughput sequencing data for each orchid species and four non-orchid Asparagales species (i.e. *Molineria capitulata*, *Hypoxis hemerocallidea*, *Lanaria larata*, *Borya sphaerocephala*) were retrieved from NCBI Sequence Read Archive (SRA, http://www.ncbi.nlm.nih.gov/sra). Raw sequence reads were first downloaded using the SRA Toolkit *fastq-dump* v2.10.8 according to their designated accession numbers. Next, adaptor removal, sliding window trimming, quality and length filtering, and base correction of the raw paired-end (PE) reads were performed with fastp v0.20.0 (Chen et al., 2018) with default settings except for the options: *l = 40* (minimum length of post-processed reads to be 40bp) and *-m* (merging of PE reads enabled). For transcriptome-related datasets, post-processed merged and unmerged PE reads were parsed to Trinity v2.8.5 (Haas et al., 2013) for *de novo* assembly using the *--single* and *--run_as_paired* option. All other assembly settings were kept as default.

Selection of accurate gene/transcript models and functional annotation

For each assembled transcriptome, resolution of sequence redundancy and classification of transcripts were achieved with the EvidentialGene *tr2aacds* pipeline (http://arthropods.eugenes.org/EvidentialGene/) using a combination of tools such as CD-HIT v4.6.8 (Li and Godzik, 2006), BLAST v2.2.29 (Camacho et al., 2009), and exonerate (Slater and Birney, 2005). Transcripts classified as 'okaysets' that satisfy various filter and quality metrics were retained for further downstream phylogenetic analysis. They include transcripts annotated as *main* – primary transcript with alternates, *alt* – alternates of *main*, and *noclass* – primary without alternates. Gene space completeness was estimated by Benchmarking Universal Single-Copy Orthologs (BUSCO) using the BUSCO v3 (Waterhouse et al., 2018) with the lineage database (*-l*) and assessment mode (*-m*) set to embryophyta_odb10 and genome, respectively.

Identification of phylogenetic-informed orthogroups at various phylogenetic depths

Inference of phylogenetic-informed orthogroups using OrthoFinder v2.5.2 (Emms and Kelly, 2019) with the multiple sequence alignment (MSA) workflow. Translated protein sequences of the EvidentialGene okaysets were used as input. In the customizable MSA workflow (*-M msa* option enabled), orthogroups were first inferred using an all-versus-all DIAMOND (Buchfink et al., 2014) search with the option: *-S diamond_ultra_sens* and *-I 1.3* enabled and the resulting sequence similarity scores were normalized for gene length bias and phylogenetic distance internally. Selection of orthogroups that are strictly single-copy in all target species (i.e. strict single-copy orthogroups) was first conducted. In cases where < 1,000 single-copy orthogroups were found, a relaxation heuristic is invoked to allow orthogroups that are single-copy for a proportion (p%) of species (i.e. relaxed single-copy orthogroups). A brief formalised procedure for determining a suitable value of *p%* provided by OrthoFinder is as follows: 1. Identify *n* (the number of orthogroups with exactly one gene in *s* species), where *s* is initially equal to *S* (the total number of species). If $n \geq 1000$, use all orthogroups and proceed to step 3. 2. Else, if $n < 1{,}000$, the minimum fraction of single-copy species

required is then progressively relaxed (i.e. set $s = S - 1, 2, 3 \ldots S$ and recalculate n, the number of orthogroups now with at least s species single-copy) to identify more orthogroups as long as that the proportional increase in orthogroups is > 2 times the proportional decrease in minimum fraction of species or until 1,000 orthogroups are identified. By default, up to one species having gene duplicates within each orthogroup is tolerated, however, we tested whether having up to 3 species having gene duplicates were also feasible for phylogenetic inference. 3. Perform amino acid MSA on selected orthogroups (strict, and relaxed where applicable) using MAFFT v7 (Katoh and Standley, 2013) with the L-INS-I option. 4. Perform light trimming of the MSA if a column consist > 90% gaps and satisfies these two criteria: No MSA will be trimmed to < 500 amino acids and no more than 25% of non-gap characters can be trimmed from the alignment. Else, if either condition is not satisfied, the percentage of gaps in removed columns is gradually increased beyond 90% until all conditions are met (Emms and Kelly, 2019).

Species and gene tree inference

Concatenation/supermatrix maximum-likelihood (ML) species tree was inferred using IQ-TREE v2 (Minh et al., 2020b) with the options: *-p* or *-S* (input folder containing amino acid MSAs from analysis options 1 or 2), *-m* MFP+MERGE, *-B* 1000, and *-alrt* 1000. With these selected options, IQ-TREE loads all amino acid MSAs and concatenates them into a supermatrix (*-p*), finds the best-fit partitioning scheme and substitution model as determined by ModelFinder (Kalyaanamoorthy et al., 2017) and performs tree search (*-m* MFP+MERGE), and measures branch support using ultrafast bootstrap (UFBoot; Hoang et al., 2018) and SH-like approximate likelihood ratio test (SH-aLRT; Guindon et al., 2010) with 1000 replicates (*-B* and *-alrt* 1000). Additionally, only general amino acid models were considered (*-msub* nuclear). Model selection and tree inference was performed separately for each amino acid MSA to obtain the bootstrapped ML gene trees with the options: *-S* (input folder containing amino acid MSAs), *-m* MFP+MERGE, *-B* 1000, and *-msub* nuclear. Bootstrapped ML gene trees were used for i. gene concordance factor (gCF%) estimation of concatenated/supermatrix ML species trees (Minh et al., 2020a) and ii. multispecies coalescent-based species tree inference using ASTRAL-MP (Yin et al., 2019), a multi-parallel implementation of ASTRAL-III (Zhang et al., 2018), with default parameters. For ASTRAL analysis, input bootstrapped ML gene trees were also contracted at various support thresholds (i.e. UFBoot ≤ 0, 5, 10, 15, 20, 25, 33, 50%) to determine the optimal threshold that maximizes the normalized quartet score of resulting ASTRAL species trees. The default branch length (i.e. in coalescent units) and support metric (i.e. local posterior probabilities) were used. Visualisation and comparative analysis of phylogenetic trees were achieved using iTOL v4 (Letunic and Bork, 2019) and ggtree (Yu et al., 2017).

REFERENCES

Buchfink, B., Xie, C., and Huson, D. H. (2014). Fast and sensitive protein alignment using DIAMOND. *Nat. Methods* 12, 59–60. doi:10.1038/nmeth.3176.

Camacho, C., Coulouris, G., Avagyan, V., Ma, N., Papadopoulos, J., Bealer, K., et al. (2009). BLAST plus : architecture and applications. *BMC Bioinformatics* 10, 421. doi:Artn 421\nDoi 10.1186/1471-2105-10-421.

Chao, Y.-T., Yen, S.-H., Yeh, J.-H., Chen, W.-C., and Shih, M.-C. (2017). Orchidstra 2.0 — A transcriptomics resource for the orchid family. *Plant Cell Physiol.* 58, pcw220. doi:10.1093/pcp/pcw220.

Chen, S., Zhou, Y., Chen, Y., and Gu, J. (2018). Fastp: An ultra-fast all-in-one FASTQ preprocessor. *Bioinformatics* 34, i884–i890. doi:10.1093/bioinformatics/bty560.

Cheon, S., Zhang, J., and Park, C. (2020). Is Phylotranscriptomics as Reliable as Phylogenomics? *Mol. Biol. Evol.* 37, 3672–3683. doi:10.1093/molbev/msaa181.

Deng, H., Zhang, G. Q., Lin, M., Wang, Y., and Liu, Z. J. (2015). Mining from transcriptomes: 315 single-copy orthologous genes concatenated for the phylogenetic analyses of Orchidaceae. *Ecol. Evol.* 5, 3800–3807. doi:10.1002/ece3.1642.

Emms, D. M., and Kelly, S. (2015). OrthoFinder: solving fundamental biases in whole genome comparisons dramatically improves orthogroup inference accuracy. *Genome Biol.* 16, 157. doi:10.1186/s13059-015-0721-2.

Emms, D. M., and Kelly, S. (2018). STAG: Species Tree Inference from All Genes. doi:doi: https://doi.org/10.1101/267914.

Emms, D. M., and Kelly, S. (2019). OrthoFinder: Phylogenetic orthology inference for comparative genomics. *Genome Biol.* 20, 238. doi:10.1186/s13059-019-1832-y.

Eserman, L. A., Thomas, S. K., Coffey, E. E. D., and Leebens-Mack, J. H. (2021). Target sequence capture in orchids: Developing a kit to sequence hundreds of single-copy loci. *Appl. Plant Sci.* 9, e11416. doi:10.1002/aps3.11416.

Guindon, S., Dufayard, J. F., Lefort, V., Anisimova, M., Hordijk, W., and Gascuel, O. (2010). New algorithms and methods to estimate maximum-likelihood phylogenies: Assessing the performance of PhyML 3.0. *Syst. Biol.* 59, 307–321. doi:10.1093/sysbio/syq010.

Guo, Y.-Y., Zhang, Y.-Q., Zhang, G.-Q., Huang, L.-Q., and Liu, Z.-J. (2018). Comparative transcriptomics provides insight into the molecular basis of species diversification of section Trigonopedia (*Cypripedium*) on the Qinghai-Tibetan Plateau. *Sci. Rep.* 8, 11640. doi:10.1038/s41598-018-30147-9.

Guo, Y. Y., Luo, Y. B., Liu, Z. J., and Wang, X. Q. (2012). Evolution and biogeography of the slipper orchids: Eocene vicariance of the conduplicate genera in the old and new world tropics. *PLoS One* 7, e38788. doi:10.1371/journal.pone.0038788.

Haas, B. J., Papanicolaou, A., Yassour, M., Grabherr, M., Blood, P. D., Bowden, J., et al. (2013). De novo transcript sequence reconstruction from RNA-seq using the Trinity platform for reference generation and analysis. *Nat. Protoc.* 8, 1494–1512. doi:10.1038/nprot.2013.084.

Hoang, D. T., Chernomor, O., Von Haeseler, A., Minh, B. Q., and Vinh, L. S. (2018). UFBoot2: Improving the ultrafast bootstrap approximation. *Mol. Biol. Evol.* 35, 518–522. doi:10.1093/molbev/msx281.

Hölzer, M., and Marz, M. (2019). De novo transcriptome assembly: A comprehensive cross-species comparison of short-read RNA-Seq assemblers. *Gigascience* 8, 1–16. doi:10.1093/gigascience/giz039.

Huang, C. H., Sun, R., Hu, Y., Zeng, L., Zhang, N., Cai, L., et al. (2016). Resolution of

brassicaceae phylogeny using nuclear genes uncovers nested radiations and supports convergent morphological evolution. *Mol. Biol. Evol.* 33, 394–412. doi:10.1093/molbev/msv226.

Kalyaanamoorthy, S., Minh, B. Q., Wong, T. K. F., Von Haeseler, A., and Jermiin, L. S. (2017). ModelFinder: Fast model selection for accurate phylogenetic estimates. *Nat. Methods* 14, 587–589. doi:10.1038/nmeth.4285.

Katoh, K., and Standley, D. M. (2013). MAFFT multiple sequence alignment software version 7: Improvements in performance and usability. *Mol. Biol. Evol.* 30, 772–780. doi:10.1093/molbev/mst010.

Kim, Y. K., Jo, S., Cheon, S. H., Joo, M. J., Hong, J. R., Kwak, M., et al. (2020). Plastome evolution and phylogeny of Orchidaceae, with 24 new sequences. *Front. Plant Sci.* 11, 22. doi:10.3389/fpls.2020.00022.

Kumar, S., Filipski, A. J., Battistuzzi, F. U., Kosakovsky Pond, S. L., and Tamura, K. (2012). Statistics and truth in phylogenomics. *Mol. Biol. Evol.* 29, 457–472. doi:10.1093/molbev/msr202.

Lee, T. H., Tang, H., Wang, X., and Paterson, A. H. (2013). PGDD: A database of gene and genome duplication in plants. *Nucleic Acids Res.* 41, 1152–1158. doi:10.1093/nar/gks1104.

Leebens-Mack, J. H., Barker, M. S., Carpenter, E. J., Deyholos, M. K., Gitzendanner, M. A., Graham, S. W., et al. (2019). One thousand plant transcriptomes and the phylogenomics of green plants. *Nature* 574, 679–685. doi:10.1038/s41586-019-1693-2.

Letunic, I., and Bork, P. (2019). Interactive Tree of Life (iTOL) v4: Recent updates and new developments. *Nucleic Acids Res.* 47, 256–259. doi:10.1093/nar/gkz239.

Li, W., and Godzik, A. (2006). Cd-hit: A fast program for clustering and comparing large sets of protein or nucleotide sequences. *Bioinformatics* 22, 1658–1659. doi:10.1093/bioinformatics/btl158.

Li, Y. X., Li, Z. H., Schuitman, A., Chase, M. W., Li, J. W., Huang, W. C., et al. (2019). Phylogenomics of Orchidaceae based on plastid and mitochondrial genomes. *Mol. Phylogenet. Evol.* 139, 106540. doi:10.1016/j.ympev.2019.106540.

Minh, B. Q., Hahn, M. W., and Lanfear, R. (2020a). New Methods to Calculate Concordance Factors for Phylogenomic Datasets. *Mol. Biol. Evol.* 37, 2727–2733. doi:10.1093/molbev/msaa106.

Minh, B. Q., Schmidt, H. A., Chernomor, O., Schrempf, D., Woodhams, M. D., Von Haeseler, A., et al. (2020b). IQ-TREE 2: New models and efficient methods for phylogenetic inference in the genomic era. *Mol. Biol. Evol.* 37, 1530–1534. doi:10.1093/molbev/msaa015.

Pérez-Escobar, O. A., Dodsworth, S., Bogarín, D., Bellot, S., Balbuena, J. A., Schley, R. J., et al. (2021). Hundreds of nuclear and plastid loci yield novel insights into orchid relationships. *Am. J. Bot.* 108, 1166–1180. doi:10.1002/ajb2.1702.

Piñeiro Fernández, L., Byers, K. J. R. P., Cai, J., Sedeek, K. E. M., Kellenberger, R. T., Russo, A., et al. (2019). A phylogenomic analysis of the floral transcriptomes of sexually deceptive and rewarding European orchids, *Ophrys* and *Gymnadenia*. *Front. Plant Sci.* 10, 1553. doi:10.3389/fpls.2019.01553.

Qiao, X., Li, Q., Yin, H., Qi, K., Li, L., Wang, R., et al. (2019). Gene duplication and evolution in recurring polyploidization-diploidization cycles in plants. *Genome Biol.* 20, 1–23. doi:10.1186/s13059-019-1650-2.

Serna-Sánchez, M. A., Pérez-Escobar, O. A., Bogarín, D., Torres-Jimenez, M. F., Alvarez-Yela, A. C., Arcila-Galvis, J. E., et al. (2021). Plastid phylogenomics resolves ambiguous relationships within the orchid family and provides a solid timeframe for biogeography and macroevolution. *Sci. Rep.* 11, 6858. doi:10.1038/s41598-021-83664-5.

Slater, G. S. C., and Birney, E. (2005). Automated generation of heuristics for biological sequence comparison. *BMC Bioinformatics* 6, 1–11. doi:10.1186/1471-2105-6-31.

Tsai, C. C., Chiang, Y. C., Huang, S. C., Chen, C. H., and Chou, C. H. (2010). Molecular phylogeny of *Phalaenopsis* Blume (Orchidaceae) on the basis of plastid and nuclear DNA. *Plant Syst. Evol.* 288, 77–98. doi:10.1007/s00606-010-0314-1.

Unruh, S. A., McKain, M. R., Lee, Y. I., Yukawa, T., McCormick, M. K., Shefferson, R. P., et al. (2018). Phylotranscriptomic analysis and genome evolution of the Cypripedioideae (Orchidaceae). *Am. J. Bot.* 105, 631–640. doi:10.1002/ajb2.1047.

Waterhouse, R. M., Seppey, M., Simao, F. A., Manni, M., Ioannidis, P., Klioutchnikov, G., et al. (2018). BUSCO applications from quality assessments to gene prediction and phylogenomics. *Mol. Biol. Evol.* 35, 543–548. doi:10.1093/molbev/msx319.

Wickett, N. J., Mirarab, S., Nguyen, N., Warnow, T., Carpenter, E., Matasci, N., et al. (2014). Phylotranscriptomic analysis of the origin and early diversification of land plants. *Proc. Natl. Acad. Sci. U. S. A.* 111, E4859-68. doi:10.1073/pnas.1323926111.

Wong, D. C. J., Amarasinghe, R., Falara, V., Pichersky, E., and Peakall, R. (2019). Duplication and selection in β-ketoacyl-ACP synthase gene lineages in the sexually deceptive *Chiloglottis* (Orchidaceace). *Ann. Bot.* 123, 1053–1066. doi:10.1093/aob/mcz013.

Xiang, Y., Huang, C. H., Hu, Y., Wen, J., Li, S., Yi, T., et al. (2017). Evolution of Rosaceae Fruit Types Based on Nuclear Phylogeny in the Context of Geological Times and Genome Duplication. *Mol. Biol. Evol.* 34, 262–281. doi:10.1093/molbev/msw242.

Yin, J., Zhang, C., and Mirarab, S. (2019). ASTRAL-MP: Scaling ASTRAL to very large datasets using randomization and parallelization. *Bioinformatics* 35, 3961–3969. doi:10.1093/bioinformatics/btz211.

Yu, G., Smith, D. K., Zhu, H., Guan, Y., and Lam, T. T. Y. (2017). ggtree: an R package for visualization and annotation of phylogenetic trees with their covariates and other associated data. *Methods Ecol. Evol.* 8, 28–36. doi:10.1111/2041-210X.12628.

Zhang, C., Rabiee, M., Sayyari, E., and Mirarab, S. (2018). ASTRAL-III: Polynomial time

species tree reconstruction from partially resolved gene trees. *BMC Bioinformatics* 19, 153. doi:10.1186/s12859-018-2129-y.

Zhang, C., Zhang, T., Luebert, F., Xiang, Y., Huang, C.-H., Hu, Y., et al. (2020). Asterid phylogenomics/phylotranscriptomics uncover morphological evolutionary histories and support phylogenetic placement for numerous whole-genome duplications. *Mol. Biol. Evol.* 37, 3188–3210. doi:10.1093/molbev/msaa160.

Zhang, G. Q., Liu, K. W., Li, Z., Lohaus, R., Hsiao, Y. Y., Niu, S. C., et al. (2017). The *Apostasia* genome and the evolution of orchids. *Nature* 549, 379–383. doi:10.1038/nature23897
