# Supplementary material for: Orchid Phylotranscriptomics: The Prospects of Repurposing Multi-Tissue Transcriptomes for Phylogenetic Analysis and Beyond
Source: Front Plant Sci. 2022 May 27;13:910362. doi: 10.3389/fpls.2022.910362 (PMC9196242; doi:10.3389/fpls.2022.910362)
Supplement: Supplementary file 3 [file Data_Sheet_2.pdf]

SUPPLEMENTARY FIGURES AND TABLES

Orchid phylotranscriptomics: The prospects of repurposing multi-tissue transcriptomes for phylogenetic analysis and beyond

Darren C.J. Wong[1*] and Rod Peakall[1]

[1]Ecology and Evolution, Research School of Biology, The Australian National University, Canberra ACT 2600, Australia

*** Correspondence:**

Darren CJ Wong

darren.wong@anu.edu.au; wongdcj@gmail.com

+61 2 6125 9892

FIGURE S1. Summary of key assembly and quality metrics of repurposed multi-tissue transcriptome from 133 target orchid species. (A) Boxplots show the length (average/median and N50) and total contig distribution in the full (blue) and filtered (red) transcriptome assemblies. (B) Boxplot shows the distribution of Benchmarking Universal Single-Copy Orthologs (BUSCO) gene content classified as complete single-copy, complete duplicated, fragmented, and missing for the final quality-filtered reassembled transcriptomes. The embryophyte lineage database encompassing 1,375 BUSCOs was used as a reference. See **supplementary table 3** for full details.

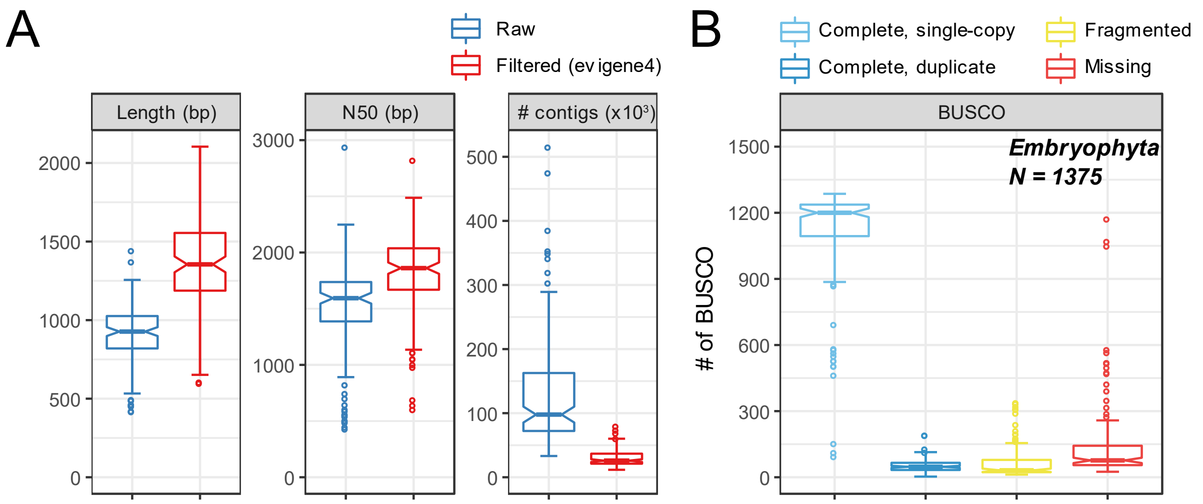

FIGURE S2. Outcomes of phylogenetic analysis across the Orchidaceae from repurposed multi-tissue transcriptomes. Tangle plots of (A) maximum-likelihood IQ-TREE and (B) shortcut coalescent ASTRAL phylogeny of 69 orchids (spanning 5 subfamilies, 13 tribes, 21 subtribes, and 48 genera) and four non-orchid species as outgroup. In (A), the corresponding ML species tree was based on partition analysis of 633 amino-acid alignments with a total of 317,221 (139,913 parsimony-informative) sites. In (B), bootstrapped ML IQ-TREE gene trees (633 total) were used ASTRAL species tree inference (See **Supplementary Methods** for details). The abbreviated species name is shown (see **Table 1** for details) and background colour indicates subfamily grouping. Background in (A), branch colour indicated gene concordance factors (gCF). Branch labels show respective gCF alongside site concordance factors sCF (i.e. g*N*/s*N*). Only branches that do not satisfy an ultrafast bootstrap (UFboot) ≥ 95% or SH-aLRT support ≥ 80% are indicated in red alongside gCF and sCF scores. Branch length indicates the total number of substitutions per site. Inset depicts the relationships of gCF, sCF, UFboot and SH-aLRT against branch length. In (B), branches receiving local posterior probability scores < 1 are labelled in red. Branch length indicates coalescent units.

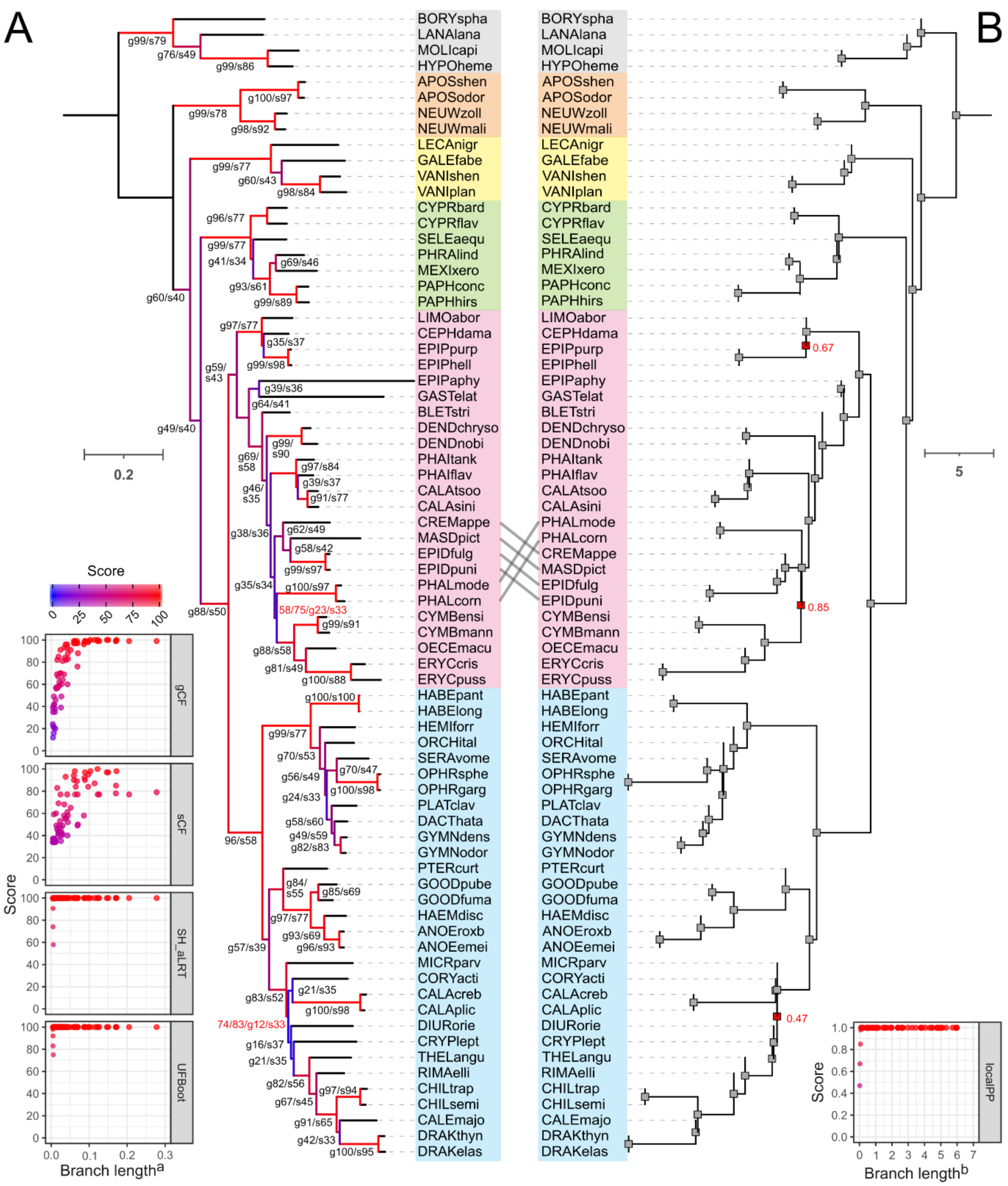
A
B
BORYspha
LANAlana
MOLIcapi
HYPOheme
APOSshen
APOSodor
NEUWzoll
NEUWmali
LECAnigr
GALEfabe
VANIshen
VANIplan
CYPRbard
CYPRflav
SELEaequ
PHRAlind
MEXIxero
PAPHconc
PAPHhirs
LIMOabor
CEPHdama
EPIPpurp
EPIPhell
EPIPaphy
GASTelat
BLETstri
DENDchryso
DENDnobi
PHALtank
PHALflav
CALAtsoo
CALAsini
CREMappe
MASDpict
EPIDfulg
EPIDpuni
PHALmode
PHALcorn
CYMBensi
CYMBmann
OECEmacu
ERYCcris
ERYCpuss
HABEpant
HABElong
HEMIforr
ORCHital
SERAvome
OPHRsphe
OPHRgarg
PLATclav
DACThata
GYMNdens
GYMNodor
PTERcurt
GOODpube
GOODfuma
HAEMdisc
ANOExoxb
ANOEemei
MICRparv
CORYacti
CALAcreb
CALAplic
DIURorie
CRYPlept
THELangu
RIMAelli
CHILtrap
CHILsemi
CALEmajo
DRAKthyn
DRAKelas
0.2
5
Score
0
25
50
75
100
gCF
sCF
SH_aLRT
UFBoot
localPP
Branch length[a]
Branch length[b]
58/75/g23/s33
74/83/g12/s33
0.67
0.85
0.47

FIGURE S3. Outcomes of Cypripedioideae subfamily phylogenetic analysis using repurposed multi-tissue transcriptomes. The Maximum-likelihood IQ-TREE based on concatenated protein sequence alignments of 19 species and *Vanilla shenzhenica* (outgroup) is shown. The abbreviated species name is shown (see **Table 1** for details) and the background colour indicates section grouping. Branch labels show respective gCF alongside site concordance factors sCF (i.e. gN/sN). All but one branch received an ultrafast bootstrap (UFboot) and SH-aLRT support of 100 and is indicated in red. Branch length indicates the total number of substitutions per site. Background colour indicates genus designation. Labels A – E indicates section Subtropica, Flabellinervia, Obtusipetala, Sinopedilum, and Trigonopedia within the genus Cypripedium. Labels 1 – 3 indicate subgenus Parvisepalum, Brachypetalum, and Paphiopedilum within the genus Paphiopedilum.

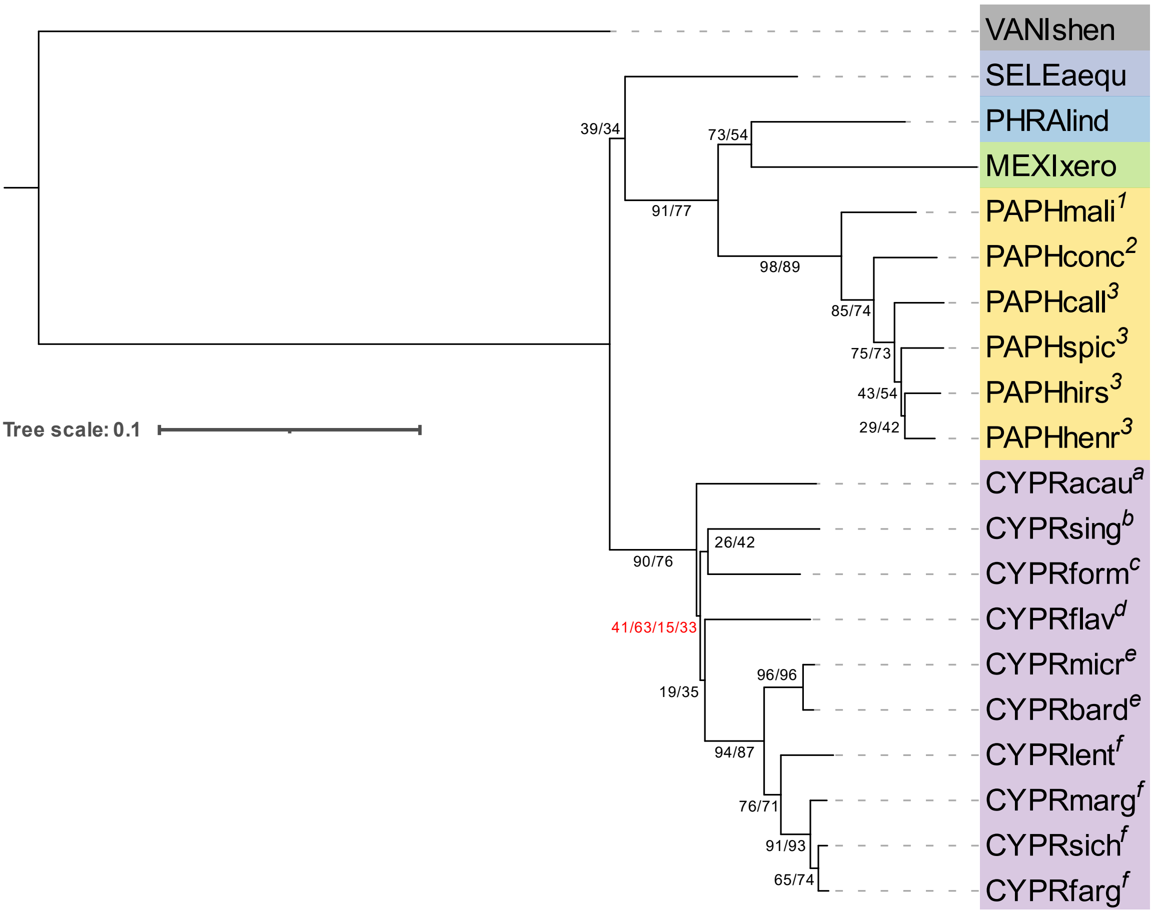

FIGURE S4. Outcomes of *Phalaenopsis* phylogenetic analysis using repurposed multi-tissue transcriptomes. The Maximum-likelihood IQ-TREE based on concatenated protein sequence alignments of 11 species and *Erycina pusilla* (outgroup) is shown. The abbreviated species name is shown (see **Table 1** for details) and background colour indicates subgenus grouping. Branch labels show respective gCF alongside site concordance factors sCF (i.e. gN/sN). All branches received an ultrafast bootstrap and SH-aLRT support of 100, thus not explicitly labelled for clarity. Background colour indicates subgenus designation (light blue, *Phalaenopsis*; light orange, *Polychilos*). Branch length indicates the total number of substitutions per site.

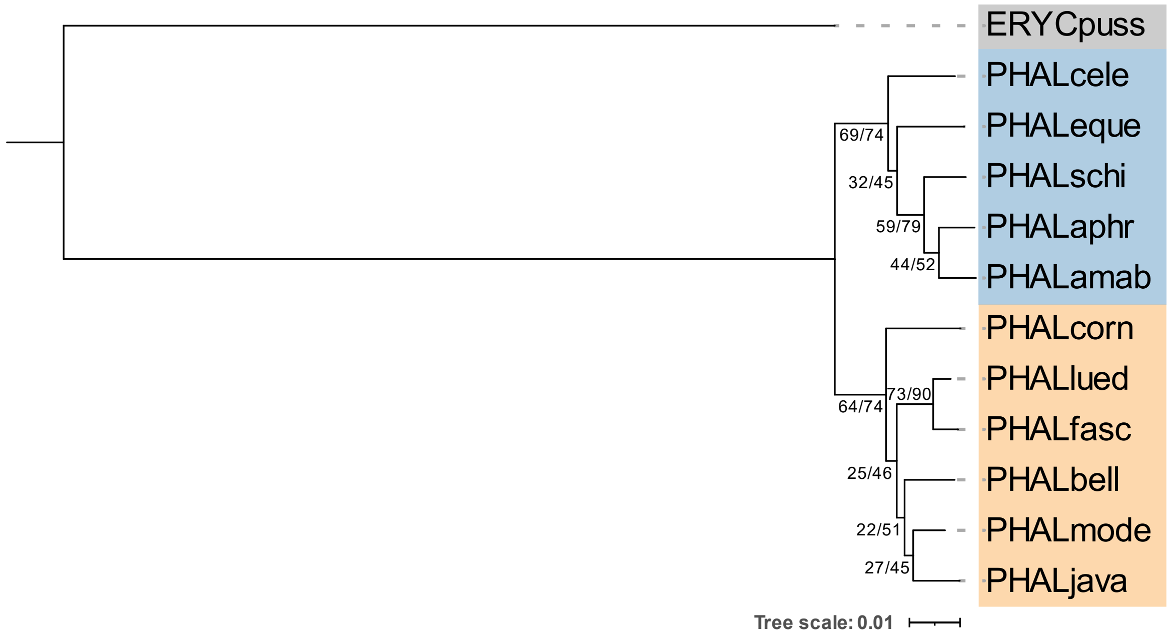

TABLE S1. Taxonomic breadth of orchid multi-tissue transcriptomes available for phylotranscritomic analysis in this study. Asterisks indicate species used for phylotranscriptomic analysis presented in **Figure 1** and **Supplementary Figure 2**. Symbol, subfamily (AP, Apostasioideae; CY, Cypripedioideae; EP, Epidendroideae; OR, Orchidoideae; VA, Vanilloideae), tribe, and subtribe designation of each species are indicated. Study type indicates the primary goal – i.e. for understanding specific biological processes (BP); phylogenomic analysis (PHY), or as a genetic resource (RES) – of sequencing the corresponding species transcriptome in the original study (see Citation column). Tissue type indicates the tissues considered for transcriptome re-assembly for phylotranscriptpmic analysis in this study (S, seed; GS, germinated seed; L, leaf; FLW, flower, P, pollinia; B, buds; M, mix tissues; SH, shoot; FLW-P, pistil; ST, stem; FLW-S, style; W, whole plant/above-ground tissues; R, root). When records of the original publication(s) are not available, the designated NCBI Bioproject accession (PRJNA#) is shown. See **Supplementary Table 2** for outgroup details.

**Species**		**Symbol**	**Subfamily**	**Tribe**	**Subtribe**	**Study type**	**Tissue type**	**Citation**
Anoectochilus emeiensis	*	ANOEemei	OR	Cranichideae	Goodyerinae	BP	L	PRJNA496377
Anoectochilus roxburghii	*	ANOEroxb	OR	Cranichideae	Goodyerinae	BP	S, GS	Liu et al. (2015) International Journal of Molecular Sciences 16(12):30190–30203
Apostasia nipponica	*	APOSnipp	AP			PHY	L	Unruh et al. (2018) American Journal of Botany 105(4):631–640
Apostasia odorata	*	APOSodor	AP			PHY	FLW	Zhang et al. (2017) Nature 549:379–383
Apostasia shenzhenica		APOSshen	AP			PHY	P, S	Zhang et al. (2017) Nature 549:379–383
Apostasia wallichii		APOSwall	AP			RES	L, B	Chao et al. (2017) Plant Cell Physiology 58(1):e9
Bletilla striata	*	BLETstri	EP	Arethuseae	Coelogyninae	BP	M	Xu et al. (2018a) PLoS ONE 13(10): e0205954

Caladenia actensis		CALAacte	OR	Diurideae	Caladeniinae	RES	FLW	Peakall et al. (2021) Molecular Ecology Resources 21(4):1118–1140
Caladenia attingens		CALAatti	OR	Diurideae	Caladeniinae	RES	FLW	Peakall et al. (2021) Molecular Ecology Resources 21(4):1118–1140
Caladenia crebra	*	CALAcreb	OR	Diurideae	Caladeniinae	RES	FLW	Peakall et al. (2021) Molecular Ecology Resources 21(4):1118–1140
Caladenia denticulata		CALAdent	OR	Diurideae	Caladeniinae	RES	L	Peakall et al. (2021) Molecular Ecology Resources 21(4):1118–1140
Caladenia longicauda		CALAlong	OR	Diurideae	Caladeniinae	RES	L	Peakall et al. (2021) Molecular Ecology Resources 21(4):1118–1140
Caladenia moora		CALAmoor	OR	Diurideae	Caladeniinae	RES	L	Peakall et al. (2021) Molecular Ecology Resources 21(4):1118–1140
Caladenia plicata	*	CALAplic	OR	Diurideae	Caladeniinae	BP, RES	L	Xu et al. (2017) Current Biology 27, 1867–1877, Peakall et al. (2021) Molecular Ecology Resources 21(4):1118–1140
Calanthe hoshii		CALAhosh	EP	Collabieae		BP	NS	Hamabata et al. (2019) Communications Biology 2:244
Calanthe masuca		CALAmasu	EP	Collabieae		BP	L	Hu et al. (2018) BMC Genomics 19: 800
Calanthe sinica	*	CALAsini	EP	Collabieae		BP	L	Hu et al. (2018) BMC Genomics 19: 800

Calanthe triplicata		CALAtrip	EP	Collabieae		BP	NS	Hamabata et al. (2019) Communications Biology 2:244
Calanthe tsoongiana	*	CALAtsoo	EP	Collabieae		BP	S	Jiang et al. (2021) Gene 772:145355
Caleana major	*	CALEmajo	OR	Diurideae	Drakaeinae	RES	L	Peakall et al. (2021) Molecular Ecology Resources 21(4):1118–1140
Cephalanthera damasonium	*	CEPHdama	EP	Neottieae		BP	B	Lallemand et al. (2019) Plant Journal 98(5):826–841
Chiloglottis seminuda	*	CHILsemi	OR	Diurideae	Drakaeinae	BP, RES, PHY	FLW	Wong et al. (2019) Annals of Botany 123: 1053–1066, Peakall et al. (2021) Molecular Ecology Resources 21(4):1118–1140
Chiloglottis trapeziformis	*	CHILtrap	OR	Diurideae	Drakaeinae	BP, RES, PHY	L	Wong et al. (2017a) Frontiers in Plant Science 8:1260, Wong et al. (2018) Frontiers in Plant Science 9:389, Peakall et al. (2021) Molecular Ecology Resources 21(4):1118–1140, Wong et al. (2022) Frontiers in Plant Science 13:860997
Chiloglottis valida		CHILvali	OR	Diurideae	Drakaeinae	RES	FLW	Peakall et al. (2021) Molecular Ecology Resources 21(4):1118–1140
Corybas actinoflorus	*	CORYacti	OR	Diurideae	Acianthinae	RES	L	Peakall et al. (2021) Molecular Ecology Resources 21(4):1118–1140

Cremastra appendiculata	*	CREMappe	EP	Epidendreae	Calypsoinae	BP	B	Li et al. (2018) International Journal of Molecular Sciences 19(11):3354
Cryptostylis leptochila	*	CRYPlept	OR	Diurideae	Cryptostylidinae	RES	L	Peakall et al. (2021) Molecular Ecology Resources 21(4):1118–1140
Cymbidium atropurpureum		CYMBatro	EP	Cymbidieae	Cymbidiinae	BP, PHY	L	Zhang et al. (2016b) Plant Journal 86:175–185
Cymbidium ensifolium	*	CYMBensi	EP	Cymbidieae	Cymbidiinae	BP	FLW	Li et al. (2013) PLoS ONE 8:e85480
Cymbidium faberi		CYMBfabe	EP	Cymbidieae	Cymbidiinae	BP	L	Sun et al. (2016) SpringerPlus 5:1458
Cymbidium goeringii		CYMBgoer	EP	Cymbidieae	Cymbidiinae	BP	FLW	Ramya et al. (2019) BMC Plant Biology 19:337
Cymbidium kanran		CYMBkanr	EP	Cymbidieae	Cymbidiinae	BP	M	Luo et al. (2019) Indian Journal of Genetics and Plant Breeding 79:485–493, Zhou et al. (2021) Frontiers in Plant Science 12:737815
Cymbidium longibracteatum		CYMBlong	EP	Cymbidieae	Cymbidiinae	BP	L	Yu et al. (2020) Tree Genetics & Genomes 16:44
Cymbidium mannii	*	CYMBmann	EP	Cymbidieae	Cymbidiinae	BP, PHY	L	Zhang et al. (2016b) Plant Journal 86:175–185
Cymbidium sinense		CYMBsine	EP	Cymbidieae	Cymbidiinae	BP	M	Zhang et al. (2013) BMC Genomics 14:279

Cymbidium tortisepalum		CYMBtort	EP	Cymbidieae	Cymbidiinae	BP	L	Zhao et al. (2019) Russian Journal of Plant Physiology 66:618–627
Cymbidium tracyanum		CYMBtrac	EP	Cymbidieae	Cymbidiinae	BP	R	Li et al. (2020) Annals of Botany 126(2):261–275
Cypripedium acaule		CYPRacau	CY			PHY	L	Unruh et al. (2018) American Journal of Botany 105(4):631–640
Cypripedium bardolphianum	*	CYPRbard	CY			BP, PHY	L, FLW	Guo et al. (2018) Scientific Reports 8:11640
Cypripedium fargesii		CYPRfarg	CY			BP, PHY	FLW	Guo et al. (2018) Scientific Reports 8:11640
Cypripedium formosanum		CYPRform	CY			RES	B, FLW	Chao et al. (2017) Plant Cell Physiology 58(1):e9
Cypripedium lentiginosum		CYPRlent	CY			BP, PHY	L, FLW	Guo et al. (2018) Scientific Reports 8:11640
Cypripedium margaritaceum		CYPRmarg	CY			BP, PHY	L, FLW	Guo et al. (2018) Scientific Reports 8:11640
Cypripedium micranthum		CYPRmicr	CY			BP, PHY	L, FLW	Guo et al. (2018) Scientific Reports 8:11640
Cypripedium sichuanense		CYPRsich	CY			BP, PHY	L, FLW	Guo et al. (2018) Scientific Reports 8:11640
Cypripedium flavum	*	CYPRflav	CY			BP, PHY	L, FLW	Guo et al. (2018) Scientific Reports 8:11640

Cypripedium singchii		CYPRsing	CY			BP, PHY	L, FLW	Guo et al. (2018) Scientific Reports 8:11640
Dactylorhiza fuchsii		DACTfuch	OR	Orchideae	Orchidinae	BP	L	Balao et al. (2017) Molecular Ecology 26:3649–3662
Dactylorhiza hatagirea	*	DACThata	OR	Orchideae	Orchidinae	BP	L, SH	Dhiman et al. (2019) Scientific Reports 9:13133
Dactylorhiza incarnata		DACTinca	OR	Orchideae	Orchidinae	BP	L	Balao et al. (2017) Molecular Ecology 26:3649–3662
Dendrobium chrysanthum		DENDchrys	EP	Malaxideae	Dendrobiinae	BP	FLW–P	Niu et al. (2017) Frontiers in Plant Science 8:1106
Dendrobium chrysotoxum	*	DENDchryso	EP	Malaxideae	Dendrobiinae	BP, PHY	FLW–P	Zhang et al. (2021) Horticulture Research 8:183
Dendrobium huoshanense		DENDhuos	EP	Malaxideae	Dendrobiinae	BP	ST	Ma et al. (2020) RNA Biology 17(9):1223–1227
Dendrobium longicornu		DENDlong	EP	Malaxideae	Dendrobiinae	BP	FLW–S	Niu et al. (2017) Frontiers in Plant Science 8:1106
Dendrobium moniliforme		DENDmoni	EP	Malaxideae	Dendrobiinae	BP	L	Yuan et al. (2019) BMC Plant Biology 19:521
Dendrobium nobile	*	DENDnobi	EP	Malaxideae	Dendrobiinae	BP	L, FLW, R	Xu et al. (2022) Frontiers in Genetics 13:844622
Dendrobium officinale		DENDoffi	EP	Malaxideae	Dendrobiinae	BP	W	Zhang et al. (2016a) Frontiers in Plant Science 7:5
Dendrobium palpebrae		DENDpalp	EP	Malaxideae	Dendrobiinae	PHY	L	Unruh et al. (2018) American Journal of Botany 105(4):631–640

Dendrobium terminale		DENDterm	EP	Malaxideae	Dendrobiinae	BP, PHY	L	Zhang et al. (2016b) Plant Journal 86:175–185
Dendrobium wardianum		DENDward	EP	Malaxideae	Dendrobiinae	BP	L	Li et al. (2019a) Industrial Crops and Products 28:48–54
Dendrobium williamsonii		DENDwill	EP	Malaxideae	Dendrobiinae	BP	ST	Ma et al. (2020) RNA Biology 17(9):1223–1227
Diuris orientis	*	DIURorie	OR	Diurideae	Diuridinae	RES	L	Peakall et al. (2021) Molecular Ecology Resources 21(4):1118–1140
Drakaea elastica	*	DRAKelas	OR	Diurideae	Drakaeinae	PHY	L, B, FLW	Leebens-Mack et al. (2019) Nature 574:679–685
Drakaea thynniphila	*	DRAKthyn	OR	Diurideae	Drakaeinae	RES	FLW	Peakall et al. (2021) Molecular Ecology Resources 21(4):1118–1140
Epidendrum fulgens	*	EPIDfulg	EP	Epidendreae	Laeliinae	BP	R	Leal et al. (2020) BMC Plant Biology 20:554
Epidendrum puniceoluteum	*	EPIDpuni	EP	Epidendreae	Laeliinae	BP	R	Leal et al. (2020) BMC Plant Biology 20:554
Epipactis helleborine	*	EPIPhell	EP	Neottieae		BP	B, R	Suetsugu et al. (2017) Molecular Ecology 26:1652–1669, Lallemand et al. (2019) Plant Journal 98(5):826–841
Epipactis purpurata	*	EPIPpurp	EP	Neottieae		BP	B	Lallemand et al. (2019) Plant Journal 98(5):826–841
Epipogium aphyllum	*	EPIPaphy	EP	Nervilieae	Epipogiinae	BP	W	Schelkunov et al. (2018) BMC Genomics 19:602, Jąkalski et

								al., (2021) Frontiers in Plant Science 12:632033
Epipogium roseum		EPIProse	EP	Nervilieae	Epipogiinae	BP	W	Schelkunov et al. (2018) BMC Genomics 19:602
Erycina crista–galli	*	ERYCcris	EP	Cymbidieae	Oncidiinae	BP	L	Heyduk et al. (2019) Frontiers in Plant Science 9:2000
Erycina pusilla	*	ERYCpusi	EP	Cymbidieae	Oncidiinae	BP	L	Lin et al. (2016) Plant Biotechnology Journal 14:284–298
Galeola faberi	*	GALEfabe	VA	Vanilleae		PHY	FLW	Zhang et al. (2017) Nature 549:379–383
Gastrodia elata	*	GASTelat	EP	Gastrodieae		BP, PHY	B	Yuan et al. (2018) Nature Communications 9:1615
Goodyera fumata	*	GOODfuma	OR	Cranichideae	Goodyerinae	BP	L	Lin et al. (2017) Plant Journal 90(5):994–1006
Goodyera pubescens	*	GOODpube	OR	Cranichideae	Goodyerinae	BP	L	Leebens-Mack et al. (2019) Nature 574:679–685
Gymnadenia conopsea		GYMNcono	OR	Orchideae	Orchidinae	PHY	FLW	Piñeiro Fernández et al. (2019) Frontiers in Plant Science 10:1553
Gymnadenia densiflora	*	GYMNdens	OR	Orchideae	Orchidinae	PHY	FLW	Piñeiro Fernández et al. (2019) Frontiers in Plant Science 10:1553
Gymnadenia odoratissima	*	GYMNodor	OR	Orchideae	Orchidinae	PHY	FLW	Piñeiro Fernández et al. (2019) Frontiers in Plant Science 10:1553

Gymnadenia rhellicani		GYMNrhel	OR	Orchideae	Orchidinae	PHY	FLW	Piñeiro Fernández et al. (2019) Frontiers in Plant Science 10:1553
Habenaria delavayi		HABEdela	OR	Orchideae	Orchidinae	PHY	FLW	Zhang et al. (2017) Nature 549:379–383
Habenaria longidenticulata	*	HABElong	OR	Orchideae	Orchidinae	BP	L	Lin et al. (2017) Plant Journal 90(5):994–1006
Habenaria pantlingiana	*	HABEpant	OR	Orchideae	Orchidinae	BP	L	Lin et al. (2017) Plant Journal 90(5):994–1006
Haemaria discolor	*	HAEMdisc	OR	Cranichideae	Goodyerinae	PHY	L, FLW	Leebens-Mack et al. (2019) Nature 574:679–685
Hemipilia forrestii	*	HEMIforr	OR	Orchideae	Orchidinae	PHY	FLW	Zhang et al. (2017) Nature 549:379–383
Lecanorchis nigricans	*	LECAnigr	VA	Vanilleae		PHY	FLW	Zhang et al. (2017) Nature 549:379–383
Limodorum abortivum	*	LIMOabor	EP	Neottieae		BP	R	Valadares et al. (2021) Plants (Basel) 10(2):251
Masdevallia picturata	*	MASDpict	EP	Epidendreae	Pleurothallidinae	BP	L	Lin et al. (2017) Plant Journal 90(5):994–1006
Masdevallia yuangensis		MASDyuan	EP	Epidendreae	Pleurothallidinae	PHY	L, B, R	Leebens-Mack et al. (2019) Nature 574:679–685
Mexipedium xerophyticum	*	MEXIxero	CY			PHY	L	Unruh et al. (2018) American Journal of Botany 105(4):631–640
Microtis parviflora	*	MICRparv	OR	Diurideae	Prasophyllinae	RES	L	Peakall et al. (2021) Molecular Ecology Resources 21(4):1118–1140

Neuwiedia malipoensis	*	NEUWmali	AP			PHY	FLW	Zhang et al. (2017) Nature 549:379–383
Neuwiedia zollingeri	*	NEUWzoll	AP			RES	L, B, FLW	Chao et al. (2017) Plant Cell Physiology 58(1):e9
Oeceoclades maculata	*	OECEmacu	EP	Cymbidieae	Eulophiinae	BP	R	Valadares et al. (2020) Journal of Fungi (Basel) 6(3):148
Oncidium sphacelatum		ONCIspha	EP	Cymbidieae	Oncidiinae	PHY	W	Leebens-Mack et al. (2019) Nature 574:679–685
Ophrys aymoninii		OPHRaymo	OR	Orchideae	Orchidinae	PHY	FLW	Piñeiro Fernández et al. (2019) Frontiers in Plant Science 10:1553
Ophrys exaltata		OPHRexal	OR	Orchideae	Orchidinae	BP	B	Sedeek et al. (2013) PLoS ONE 8:e64621
Ophrys garganica	*	OPHRgarg	OR	Orchideae	Orchidinae	BP	B	Sedeek et al. (2013) PLoS ONE 8:e64621
Ophrys incubacea		OPHRincu	OR	Orchideae	Orchidinae	PHY	FLW	Piñeiro Fernández et al. (2019) Frontiers in Plant Science 10:1553
Ophrys insectifera		OPHRinse	OR	Orchideae	Orchidinae	PHY	FLW	Piñeiro Fernández et al. (2019) Frontiers in Plant Science 10:1553
Ophrys iricolor		OPHRiric	OR	Orchideae	Orchidinae	PHY	FLW	Piñeiro Fernández et al. (2019) Frontiers in Plant Science 10:1553
Ophrys mesaritica		OPHRmesa	OR	Orchideae	Orchidinae	PHY	FLW	Piñeiro Fernández et al. (2019) Frontiers in Plant Science 10:1553

Ophrys sphegodes	*	OPHRsphe	OR	Orchideae	Orchidinae	BP	B	Sedeek et al. (2013) PLoS ONE 8:e64621
Orchis italica	*	ORCHital	OR	Orchideae	Orchidinae	BP	FLW	De Paolo et al. (2014) PLoS ONE 9(7):e102155
Paphiopedilum callosum		PAPHcall	CY			PHY	L	Unruh et al. (2018) American Journal of Botany 105(4):631–640
Paphiopedilum concolor	*	PAPHconc	CY			BP	L, FLW, R	Li et al. (2015) Plant Molecular Biology Reporter 33:1928–1952
Paphiopedilum henryanum		PAPHhenr	CY			BP	FLW	Xu et al. (2018b) Applications in Plant Sciences 6(5): e1152
Paphiopedilum hirsutissimum	*	PAPHhirs	CY			BP	L, FLW	Li et al. (2015) Plant Molecular Biology Reporter 33:1928–1952
Paphiopedilum malipoense		PAPHmali	CY			PHY	ST, P	Zhang et al. (2017) Nature 549:379–383
Paphiopedilum spicerianum		PAPHspic	CY			BP	PR	Fang et al. (2021) Plant Physiology and Biochemistry 167:1024–1034
Phaius flavus	*	PHAIflav	EP	Collabieae			FLW	Zhang et al. (2022) PeerJ 10:e13106
Phaius tankervilliae	*	PHAItank	EP	Collabieae		BP	S	Lee et al. (2018) Frontiers in Plant Science 9:1043.
Phalaenopsis amabilis		PHALamab	EP	Vandeae	Aeridinae	BP	FLW	Meng et al. (2020) Journal of Plant Growth Regulation 39:823–840

Phalaenopsis aphrodite		PHALaphr	EP	Vandeae	Aeridinae	RES, BP	L, FLW	Chao et al. (2017) Plant Cell Physiology 58(1): e9, Chao et al. (2018) Plant Biotechnology Journal 16:2027–2041
Phalaenopsis bellina		PHALbell	EP	Vandeae	Aeridinae	RES, BP	L, FLW	Chao et al. (2017) Plant Cell Physiology 58(1):e9; Chuang et al. (2018) Journal of Experimental Botany 69(18):4363–4377, Huang et al. (2021) Frontiers in Plant Science 12:700958
Phalaenopsis celebensis		PHALcele	EP	Vandeae	Aeridinae		L, FLW	PRJNA540261
Phalaenopsis cornu–cervi	*	PHALcorn	EP	Vandeae	Aeridinae		FLW	PRJNA540261
Phalaenopsis equestris		PHALeque	EP	Vandeae	Aeridinae	RES, BP	L, B	Cai et al. (2015) Nature Genetics 47(1):65–72
Phalaenopsis fasciata		PHALfasc	EP	Vandeae	Aeridinae		L, FLW	PRJNA540261
Phalaenopsis javanica		PHALjava	EP	Vandeae	Aeridinae		L, FLW	PRJNA540261
Phalaenopsis lueddemanniana		PHALlued	EP	Vandeae	Aeridinae	RES	L, FLW	Chao et al. (2017) Plant Cell Physiology 58(1):e9
Phalaenopsis modesta	*	PHALmode	EP	Vandeae	Aeridinae	RES	B, FLW	Chao et al. (2017) Plant Cell Physiology 58(1):e9
Phalaenopsis schilleriana		PHALschi	EP	Vandeae	Aeridinae	RES	B, FLW	Chao et al. (2017) Plant Cell Physiology 58(1):e9

Phragmipedium lindleyannum	*	PHRAlind	CY			PHY	L	Unruh et al. (2018) American Journal of Botany 105(4):631–640
Platanthera clavellata	*	PLATclav	OR	Orchideae	Orchidinae	PHY	L	Leebens-Mack et al. (2019) Nature 574:679–685
Pterostylis curta	*	PTERcurt	OR	Cranichideae	Pterostylidinae	RES	L	Peakall et al. (2021) Molecular Ecology Resources 21(4):1118–1140
Rimacola elliptica	*	RIMAelli	OR	Diurideae	Megastylidinae	RES	L	Peakall et al. (2021) Molecular Ecology Resources 21(4):1118–1140
Selenipedium aequinoctiale	*	SELEaequ	CY			PHY	L	Unruh et al. (2018) American Journal of Botany 105(4):631–640
Serapias vomeracea	*	SERAvome	OR	Orchideae	Orchidinae	BP	PR	Fochi et al. (2017) New Phytologist 213:365–379
Thelymitra angustifolia	*	THELangu	OR	Diurideae	Thelymitrinae	RES	L	Peakall et al. (2021) Molecular Ecology Resources 21(4):1118–1140
Vanilla planifolia	*	VANIplan	VA	Vanilleae		RES	L, ST	Rao et al. (2014) BMC Genomics 15:964, Hu et al. (2019) Scientific Reports 9:3416
Vanilla shenzhenica	*	VANIshen	VA	Vanilleae		RES	L, ST	Zhang et al. (2017) Nature 549:379–383

TABLE S2. Taxonomic breadth of non-orchid outgroups used for phylotranscritomic analysis in this study. Symbol, plant family designation, tissues analysed for phylotranscriptpmic analysis (i.e. L, leaf; FLW, flower), and the relevant citations are indiciated.

**Species**	**Symbol**	**Family**	**Tissue type**	**Citation**
Borya sphaerocephala	BORYspha	Boryaceae	L	Unruh et al. (2018) American Journal of Botany 105(4):631–640
Lanaria larata	LANAlara	Lanariaceae	L	Unruh et al. (2018) American Journal of Botany 105(4):631–640
Hypoxis hemerocallidea	HYPOheme	Hypoxidaceae	L	Unruh et al. (2018) American Journal of Botany 105(4):631–640
Molineria capitulata	MOLIcapi	Hypoxidaceae	FLW,L	Zhang et al. (2017) Nature 549:379–383
